# Defining hip osteoarthritis feature prevalence, severity, and change using the Scoring of Hip Osteoarthritis with MRI (SHOMRI)

**DOI:** 10.1007/s00256-024-04628-0

**Published:** 2024-03-09

**Authors:** Joshua J. Heerey, Richard B. Souza, Thomas M. Link, Johanna Luitjens, Felix Gassert, Joanne L. Kemp, Mark J. Scholes, Kay M. Crossley

**Affiliations:** 1 La Trobe Sport and Exercise Medicine Research Centre, School of Allied Health, Human Services and Sport, La Trobe University, Bundoora, Australia; 2 Department of Radiology and Biomedical Imaging, University of California-San Francisco, San Francisco, CA USA; 3 Department of Physical Therapy and Rehabilitation Science, University of California-San Francisco, San Francisco, CA USA

**Keywords:** MRI, Hip osteoarthritis, Semi-quantitative, Cohort

## Abstract

**Objective:**

To define the reporting of Scoring Hip Osteoarthritis with MRI (SHOMRI) feature prevalence and severity, and to develop criteria to monitor feature change in longitudinal investigations.

**Methods:**

Twenty-five participants (50 hips) of the femoroacetabular impingement and hip osteoarthritis cohort study underwent baseline and 2-year follow-up 3 T hip MRIs. Eight hip OA features were assessed using the SHOMRI. All MRIs were read paired with knowledge of timepoint by two blinded musculoskeletal radiologists. We provide definitions to report SHOMRI feature prevalence, severity, and longitudinal change.

**Results:**

We report clear definitions for SHOMRI feature prevalence, severity, and change. When we applied the definitions to the studied cohort, we could detect the prevalence, severity, and change of hip OA features. For example, 88% of hips had labral tears (34% graded as severe tears) and 76% had cartilage defects (42% graded as full thickness). Over 70% of hips had feature change over 2 years, highlighting the sensitivity of SHOMRI definitions to assess longitudinal change of hip OA features. Intra-reader reliability was almost perfect (weighted (w)-kappa 0.86 to 1.00), with inter-reader reliability substantial to almost perfect (w-kappa 0.80 to 1.00).

**Conclusion:**

This study is the first to provide definitions to report SHOMRI feature prevalence, severity, and change. The proposed definitions will enable comparison between hip MRI studies and improve our understanding of hip OA pathogenesis.

**Supplementary Information:**

The online version contains supplementary material available at 10.1007/s00256-024-04628-0.

## Introduction

Semi-quantitative magnetic resonance imaging (MRI) scoring systems have improved our understanding of osteoarthritis (OA) pathogenesis [1]. Most advancements have been made in knee OA, with a paucity of research undertaken in other affected joints [2]. For hip OA, three semi-quantitative MRI measures have been developed: the Hip Osteoarthritis MRI Scoring System [3], the Hip Inflammation MRI Scoring System [4], and the Scoring Hip Osteoarthritis with MRI (SHOMRI) [5]. The SHOMRI is a valid and reliable grading system that evaluates 8 different hip OA features including articular cartilage loss, bone marrow lesions (BMLs), subchondral cysts, acetabular labrum, paralabral cysts, intra-articular bodies, effusion-synovitis, and ligamentum teres abnormalities [5, 6]. It has also been used in several different populations to evaluate OA feature prevalence [7–9] and progression [10].

In the original technical paper by Lee et al. [5], the authors provided explanations for OA feature grading. However, criteria for reporting OA features (i.e., prevalence and severity) and change were not discussed. With increasing use of the SHOMRI in hip OA research and our evolving understanding of disease pathogenesis, we believe it is pertinent to advance the original SHOMRI definitions by providing new criteria for feature reporting and change where appropriate. Providing clear definitions—as has been done for knee OA [11, 12]—will facilitate consistent reporting between studies and progress our understanding of hip OA development.

The first aim of this report was to rigorously define reporting of SHOMRI hip OA feature prevalence and severity, and use these definitions in a sample of 50 hips from an ongoing prospective cohort study of young adults with hip and/or groin pain participating in high-impact physical activity (Australian football or soccer) [13]. The second aim was to develop criteria to monitor change over a period of 2 years and describe the findings in the same cohort. This report provides standardized criteria that should be applied to MRI-based semi-quantitative hip grading in clinical patient cohorts.

## Methods

### Study design

This study used a consecutive sample of participants from the femoroacetabular impingement and hip osteoarthritis cohort (FORCe) study. Briefly, the FORCe study aims to investigate changes in hip joint structures using MRI over a 2-year period in 184 symptomatic (hip and/or groin pain) men and women participating in football (Australian football or soccer) who were free of radiographic hip OA [13]. In the present study, we used the first 25 participants (50 hips) who completed baseline and 2-year follow-up hip MRIs. In addition to the eligibility criteria used for the larger FORCe study [8, 13, 14], participants were required to have hip MRIs that included the necessary sequences to enable completion of the SHOMRI assessment. Participants were recruited between August 2015 and October 2018 from sporting clubs or organisations and via online or print advertising in Melbourne and Brisbane, Australia. This study had ethics approval (La Trobe University Human Ethics Committee [HEC15-019 and HEC16-045] and the University of Queensland Human Ethics Committee [2015000916 and 2016001694], and all participants provided written informed consent.

### Magnetic resonance imaging acquisition

At baseline and 2-year follow-up, each participant completed a 3.0 T MRI (Phillips Ingenia, The Netherlands) with a 32-channel torso coil placed over the hips and pelvis. Positioning aids were used to maintain each hip in internal rotation and neutral abduction/adduction, with right and left hips imaged independently. The MRI protocol included 2D proton density (PD) spectral attenuated inversion recovery (SPAIR) sequences in a coronal, sagittal, and oblique axial orientation (Table 1).

**Table 1:** Hip magnetic resonance imaging protocol

MRI sequence	Coronal PD SPAIR	Sagittal PD SPAIR	Oblique axial PD SPAIR
Field of view (mm)	170 × 170	150 × 150	170 × 170
Slice thickness (mm)	2.5	2.5	2.5
Slice gap (mm)	1.5	1	1.5
Repetition time (ms)	2700	2675	3500
Echo time (ms)	25	25	25
Voxel size (mm)	0.70 × 0.70 × 2.5	0.7 × 0.75 × 2.5	0.75 × 0.75 × 2.5
Acquisition time (min:s)	3:17	4:18	2:35

### Development of SHOMRI definitions

To create the SHOMRI definitions, the authorship team (JJH, RBS, TML, JLK, MJS and KMC) met on two occasions to thoroughly discuss the SHOMRI scoring system and previously used semi-quantitative MRI definitions of feature prevalence, severity, and change [11, 12]. After the first meeting, the lead author (JJH) created a set of definitions which were subsequently reviewed by all authors. At the second meeting, the final definitions were discussed and agreed upon.

### SHOMRI feature assessment

Two musculoskeletal radiologists (FG and JL, 4- and 3-year experience) blinded to radiographic and clinical findings evaluated all baseline and follow-up MRIs. Both radiologists were trained by a senior musculoskeletal radiologist (TML) with > 25-year experience in semi-quantitative MRI assessment. All MRIs were read paired with knowledge of timepoint (baseline or follow-up) to improve reader reliability and sensitivity to feature OA change [12]. If discrepancies in scoring occurred, a consensus read was performed with the senior musculoskeletal radiologist. To determine intra-reader reliability, each radiologist completed SHOMRI scoring in 20 randomly selected MRIs, re-read 2 weeks after the initial scoring. For inter-reader reliability, each reader completed scoring on 50 MRIs.

### SHOMRI feature explanations

We have previously described the SHOMRI scoring system and explanations for feature grading [5, 8]. In short, eight OA features were assessed (Table 2), including articular cartilage loss (scored 0–2), BMLs (scored 0–3), subchondral cysts (scored 0–2), acetabular labrum (scored 0–5), paralabral cysts (scored present or absent), intra-articular bodies (scored present or absent), effusion-synovitis (scored present or absent), and ligamentum teres abnormalities (scored 0–3). Three features (articular cartilage loss, BMLs, and subchondral cysts) were evaluated in 10 subregions (six femoral and four acetabular). The acetabular labrum was evaluated in four subregions (anterior, anterosuperior, superior, posterior).

**Table 2:** SHOMRI feature explanations

SHOMRI feature	Graded	0	1	2	3	4	5	No. of subregions	Feature score
Cartilage defect	0–2	Absent	Partial-thickness	Full-thickness				10	0–20
BMLs	0–3	Absent	≤ 0.5 cm	> 0.5 to ≤ 1.5 cm	> 1.5 cm			10	0–30
Subchondral cyst	0–2	Absent	≤ 0.5 cm	> 0.5 cm				10	0–20
Labral tear	0–5	Absent*	Abnormal signal/fraying	Simple	Labrocartilage separation	Complex	Maceration	4	0–20
Paralabral cyst	Present/absent	Absent	Present					-	0–1
Loose body	Present/absent	Absent	Present					-	0–1
Effusion-synovitis	Present/absent	Absent	Present					-	0–1
Ligamentum teres tear	0–3	Absent	Abnormal signal/fraying	Partial	Complete			-	0–3

*BMLs* bone marrow lesions

^*^Can also include normal anatomical variants such as aplasia or hypoplasia

### Demographic and patient-reported outcome measures

Demographic information (age, sex, height, weight, symptom duration, football code participation) was collected. Each participant completed the International Hip Outcome Tool-33 (iHOT-33), a valid and reliable patient reported outcome measure[15] that is scored using a visual analog scale ranging from 0 (worst possible score) to 100 (best possible score) [15]. The iHOT-33 is recommended for evaluating hip-related quality of life in young to middle-aged people with hip and/or groin conditions [16].

### Statistical analysis

Data analyses were performed with Stata/IC 16.1 for Mac (StataCorp LC, College Station, TX, USA). Intra- and inter-reader reliability for OA features were determined with the weighted kappa or kappa (paralabral cysts only) statistic. Descriptive statistics were used to report participant characteristics and OA features (prevalence, severity and change).

## Results

### Participants

We included 25 of the first 29 participants (86%) from the FORCe study. Four participants were excluded as they did not undergo a hip MRI at 2-year follow-up. The mean age of the included symptomatic football players was 28 years, 32% were women, and average body mass index was 24 kg/m^2^. The average baseline iHOT-33 score was 65.9, with the median duration of symptoms being 36 months (interquartile range 24, 50) . Participant characteristics are described in full in Table 3.

**Table 3:** Demographic characteristics and patient-reported outcome measures

Demographic characteristics	
Age (years)	28.0 ± 6.4
Sex (% women)	32%
Height (m)	1.75 ± 0.01
Weight (kg)	75.4 ± 13.3
BMI (kg/m^2^)	24.3 (22, 26)
Football code (% soccer)	100%
Patient reported outcome measures	
iHOT33	65.9 ± 14.7

Values are presented as %, mean ± standard deviation or median (interquartile range) as appropriate

*BMI* body mass index, *iHOT-33* International Hip Outcome Tool-33

### Definitions for SHOMRI feature prevalence and severity

Definitions for prevalence and severity in the eight SHOMRI features are outlined in Table 4 and described in detail below.

**Table 4:** Definitions for reporting the prevalence, severity, and change of SHOMRI features

SHOMRI feature	Prevalence	Severity	Feature change
Articular cartilage defects	**Partial-thickness cartilage defect** - *Scored as present if partial-thickness (grade 1) cartilage loss is evident in one or more subregions (acetabular or femoral)* - *Location (in the 10 subregions) of partial-thickness cartilage defects is determined* **Full-thickness cartilage defect** - *Scored as present if full-thickness (grade 2) cartilage loss is evident in one or more subregions (acetabular or femoral)* - *Location (in the 10 subregions) of full-thickness cartilage defects is determined*	**Number of subregions with partial-thickness cartilage defects** - *The number subregions with partial-thickness cartilage defects is calculated (score range 0–10)* **Number of subregions with full-thickness cartilage defects** - *The number subregions with full-thickness cartilage defects is calculated (score range 0–10)* **Maximum cartilage score** - *The maximum cartilage defect score across all 10 subregions is determined (score range 0–2)* **Sum score** - *Cartilage scores for each subregion are summed to provide a total score (0–20)*	**Change in number of subregions affected by cartilage defects** - *Calculated separately for partial- and full-thickness cartilage defects* - *Difference in number of subregions with a cartilage defect (partial/full- thickness) at baseline and follow-up (score range 0–10)* **Number of subregions with cartilage defect worsening** - *Progression of cartilage defect grade from baseline to follow-up* - *The number of subregions with worsening is calculated (score range 0–10)* **Change in maximum cartilage score** - *Difference in maximum cartilage score (across 10 subregions) at baseline and follow-up (score range 0–2)* **Change in cartilage sum score** - *Difference in sum score at baseline and follow-up (score 0–20)* **Improvement** - *Cartilage defect improvement over time is not permitted in this definition*
Labral tears	**Labral tear (any)** - *Scored as present if a grade 2 or higher labral tear is present in at least one or more subregions* *- Location (in the 4 subregions) of labral tears is determined*	**Labral tear (simple)** - *Scored as present if a grade 2 or 3 labral tear is present in least one or more subregion* - *Grade 4/5 not present in any subregion* **Labral tear (severe)** - *Scored as present if a grade 4 or 5 labral tear is present in least one or more subregion* **Number of subregions with a labral tear (any)** - *The number subregions with a labral tear is calculated (score range 0–4)* **Maximum labral score** - *The maximum labral tear score across all 4 subregions is determined (score range 0–5)* **Sum score** *- Labral scores for each subregion are summed to provide a total score (0–20)*	**Change in number of subregions affected by a labral tear** - *Difference in number of subregions with a labral tear (any) at baseline and follow-up (score range 0–4)* **Number of subregions with labral tear worsening** - *Progression of labral tear grade from baseline to follow-up* - *The number of subregions with worsening is calculated (score range 0–4)* **Change in maximum labral score** - *Difference in maximum labral score (across 4 subregions) at baseline and follow-up (score range 0–5)* **Change in labral sum score** - *Difference in sum score at baseline and follow-up (score 0–20)* **Improvement** - *Labral tear improvement over time is not permitted in this definition*
Bone marrow lesions (BMLs)	**BML (any)** - *Scored as present if a grade 1 or higher BML is present in at least one or more subregions* - *Location (in the 10 subregions) of BMLs is determined*	**Number of subregions with a BML** - *The number subregions with a BML is calculated (score range: 0–10)* **Maximum BML score** - *The maximum BML score across all 10 subregions is determined (score range 0–3)* **Sum score** - *BML scores for each subregion are summed to provide a total score (0–30)*	**Change in number of subregions affected by a BML** - *Difference in number of subregions with a BML (grade 1 or higher) at baseline and follow-up (score range − 10–10)* **Number of subregions with BML worsening** - *Progression of BML grade from baseline to follow-up* - *The number of subregions with worsening is calculated (score range 0–10)* **Change in maximum BML score** - *Difference in maximum BML score (across 10 subregions) at baseline and follow-up (score range − 3–3)* **Change in BML sum score** *Difference in sum score at baseline and follow-up (score − 30–30)* **Improvement** **Number of subregions with BML improvement** *- Improvement of BML grade from baseline to follow-up* *- The number of subregions with improvement is calculated (Score range 0–10)*
Subchondral cysts	**Subchondral cysts (any)** *- Scored as present if a grade 1 or higher subchondral cyst is present in at least one or more subregions* *- Location (in the 10 subregions) of subchondral cysts is determined*	**Number of subregions with a subchondral cyst** *- The number subregions with a subchondral cyst is calculated (score range 0–10)* **Maximum subchondral cyst score** *- The maximum subchondral cyst score across all 10 subregions is determined (score range 0–2)* **Sum score** *- Subchondral cyst scores for each subregion are summed to provide a total score (0–20)*	**Change in number of subregions affected by a subchondral cyst** *- Difference in number of subregions with a subchondral cyst (grade 1 or higher) at baseline and follow-up (score range − 10–10)* **Number of subregions with subchondral cyst worsening** *- Progression of subchondral cyst grade from baseline to follow-up* *- The number of subregions with worsening is calculated (score range 0–10)* **Change in maximum subchondral cyst score** *- Difference in maximum subchondral cyst score (across 10 subregions) at baseline and follow-up (score range − 2–2)* **Change in subchondral cyst sum score** - *Difference in sum score at baseline and follow-up (score − 20–20)* **Improvement** ** Number of subregions with subchondral cyst improvement** - *Improvement of subchondral cyst grade from baseline to follow-up* - *The number of subregions with improvement is calculated (score range 0–10)*
Ligamentum teres tears	**Ligamentum teres tear (any)** - *Scored as present if a grade 2 or higher ligamentum teres tear is present*	**Ligamentum teres tear (partial-thickness)** - *Scored as present if a grade 2 ligamentum teres tear is present* **Ligamentum teres tear (full-thickness)** - *Scored as present if a grade 3 ligamentum teres tear is present*	**Change in Ligamentum teres tear** *Change in score from baseline to follow-up (score 0–3)* **Improvement** - *Ligamentum teres tear improvement over time is not permitted in this definition*
Other features (paralabral cysts/effusion synovitis/loose bodies)	- *Scored as present or absent*	- *Severity cannot be determined with SHOMRI scoring system*	**Change is determined by the presence of a new feature at follow-up (not present at baseline) or absence of feature at follow-up (present at baseline)**

#### Cartilage defects

Cartilage defects were present if cartilage loss was evident in one or more subregions (acetabular or femoral) and were further defined as either partial- (grade 1) or full-thickness (grade 2). The location and number of subregions (0 to 10) affected by a cartilage defect was reported separately for partial- and full-thickness defects. A maximum cartilage score (0–2) across all 10 subregions and sum score (0-20) was also determined.

#### Labral tears

A labral tear (any) was scored as present if a grade 2 or higher was reported at least one of the four subregions. We further defined labral tears into simple (grade 2 or 3) and severe (grade 4 or 5). Labral tear (any) prevalence was determined for each of the four subregions. Finally, the number of subregions (0–4) affected by a labral tear, the maximum score (0–5) across all subregions and sum score (0-20) were determined.

#### Bone marrow lesions and subchondral cysts

For BMLs and subchondral cysts, the feature was scored as present if a grade 1 or higher was observed in at least one or more subregions. For both features, prevalence was reported for all 10 subregions. The number of subregions affected (0–10), maximum score (BMLs  0–3/subchondral cysts 0–2) across all regions and sum score (BML 0-30/subchondral cyst 0-20) were determined.

#### Other features

Ligamentum teres tears were scored as present if a partial- (grade 2) or full-thickness tear (grade 3) was reported. Finally, paralabral cysts, loose bodies, and effusion-synovitis were scored as present or absent.

### Definitions for SHOMRI feature change over time

Definitions for change in the eight SHOMRI features are outlined in Table 4 and described in detail below.

#### Cartilage defects

**Fig. 1:**
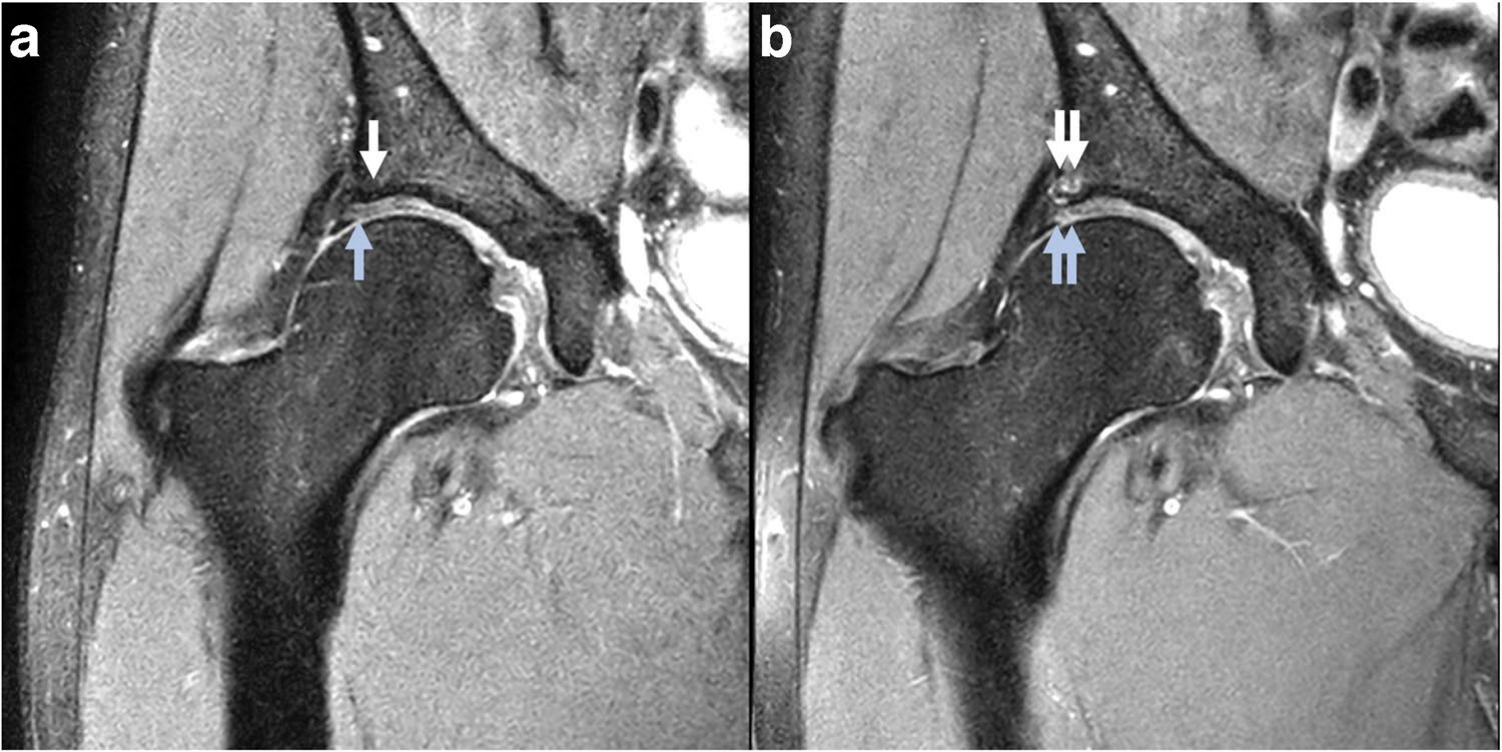
Incident cartilage defect and BML. **a** Normal subchondral bone (white arrow) and articular cartilage (blue arrow) in the superolateral subregion. **b** Two-year follow-up MRI shows incident grade 2 BML (double white arrow) and full-thickness (grade 2) acetabular cartilage defect (double blue arrow) in the superolateral subregion

Change in the number of subregions affected by cartilage defects (partial and full thickness were evaluated separately) was defined as the difference between baseline and follow-up. Cartilage defect worsening was evaluated in each subregion, and the number of subregions with worsening was determined for each hip. Overall cartilage defect worsening was defined as progression of cartilage grade in one or more subregions. Change in maximum and summed cartilage score were evaluated as the difference between baseline and follow-up scores. An example of an incident full-thickness cartilage defect after 2 years is shown in Fig. 1.

#### Labral tears

The number of new subregions affected with a labral tear (grade 2 or above) was determined by evaluating the affected subregions at baseline and follow-up. Labral tear worsening was defined as progression of labral grading in at least one or more subregions (any worsening), with the number of subregions exhibiting progression (0–4) also reported. Change in maximum and summed labral score (difference between baseline to follow-up) were determined.

#### BMLs and subchondral cysts

**Fig. 2:**
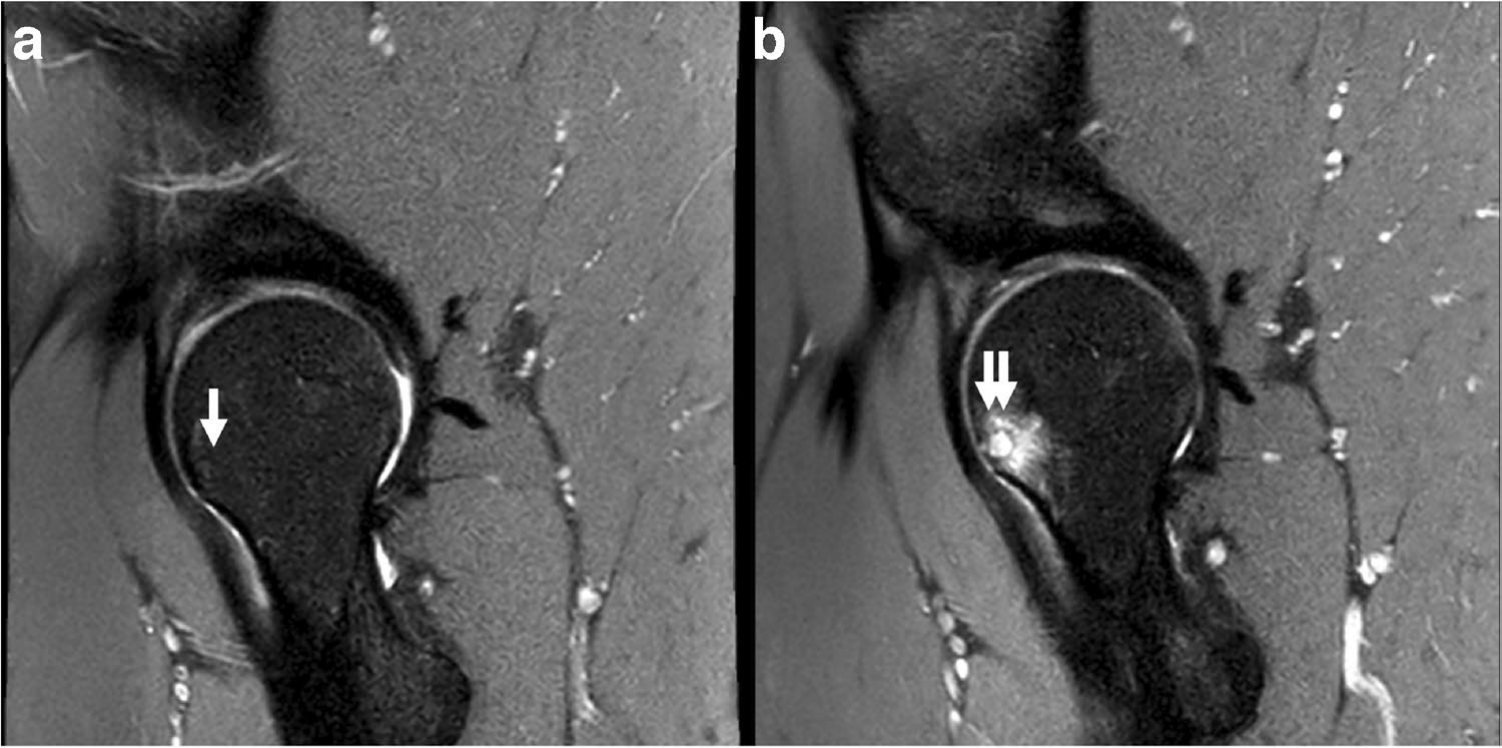
Incident subchondral cyst and BML. **a** Normal subchondral bone (white arrow) in the femoral anterior subregion. **b** Two-year follow-up MRI shows incident grade 2 subchondral cyst and grade 3 BML (double white arrow) in the femoral anterior subregion

Improvement in BMLs and subchondral cysts was allowed between baseline and follow-up. For BMLs and subchondral cysts, change in number of subregions affected (grade 1 or above) was determined by calculating the difference in affected subregions at baseline and follow-up. Feature worsening and improvement were determined for BMLs and subchondral cysts in the 10 subregions. Worsening was defined as a feature score increase (i.e., grade 1 to grade 2) in at least one subregion. Improvement was defined as feature score decrease (i.e., grade 2 to grade 1) in one or more subregions. The number of subregions with worsening and improvement was also calculated. Change (baseline to follow-up) in maximum (BML − 3 to + 3; subchondral cysts − 2 to + 2) and summed feature scores were determined for each hip. An example of an incident BML and subchondral cyst is shown in Fig. 2.

#### Other features

Worsening of ligamentum teres tears was defined as an increase of ≥ 1 in the score. For the remaining features (paralabral cysts, effusion-synovitis, and loose bodies), worsening was determined if the feature was present at follow-up but not baseline. In contrast, improvement was defined as the feature being present at baseline but not follow-up.

### Reliability

Intra- and inter-reader reliability kappa statistics results are presented in Table 5. Intra-reader reliability was almost perfect, ranging from 0.86 to 1.00 for readers 1 (FG) and 2 (JL). Inter-reader reliability ranged between 0.80 and 1.00, indicating substantial to almost perfect agreement.

**Table 5:** Intra- and inter-observer reliability

	Intra-rater reliability		Inter-rater reliability
	Reader 1 (FG)	Reader 2 (JL)	Weighted kappa (95% CI)
	Weighted kappa (95% CI)	Weighted kappa (95% CI)	
SHOMRI features			
Cartilage defect	0.86 (0.73, 1.00)	0.94 (0.80, 1.00)	0.96 (0.88, 1.00)
BMLs	—	1.00 (0.86, 1.00)	1.00 (0.93, 1.00)
Subchondral cysts	1.00 (0.87, 1.00)	1.00 (0.86, 1.00)	0.80 (0.72, 0.87)
Labral tear	0.95 (0.79, 1.00)	0.93 (0.76, 1.00)	0.90 (0.80, 1.00)
Ligamentum teres tear	1.00 (0.56, 1.00)	0.95 (0.61, 1.00)	0.89 (0.63, 1.00)
Paralabral cyst^‡^	1.00 (0.56, 1.00)	0.86 (0.42, 1.00)	0.85 (0.57, 1.00)
Loose bodies	—	—	—
Effusion-synovitis	—	—	—

Feature was not present in images assessed used for reliability assessment

*BML* bone marrow lesions

^‡^Kappa performed as feature was graded as present or absent

### SHOMRI feature prevalence and severity at baseline

Partial- and full-thickness cartilage defects were present in 76% and 42% of hips, respectively (Table 6). The number or subregions affected by cartilage defects ranged from 0 to 5, with most hips (42%) having a maximum cartilage score of 2 (Supplementary information). Labral tears were present in 88% of hips, with 34% of tears considered severe (Table 6 and Supplementary information). Over 50% of hips had 2 or more subregions affected by a labral tear (Supplementary information). Bone marrow lesions and subchondral cysts were present in 10% of hips (Table 6). No hips had evidence of effusion synovitis, loose bodies, or ligamentum teres tears, with 24% of hips having paralabral cysts (Table 6). Feature sum scores are outlined in Table 7.

**Table 6:** Baseline–hip OA feature prevalence (*n* = 50 hips)

SHOMRI feature	Outcome	Number of hips, *n* (%)
Cartilage defect	Partial thickness	38 (76%)
	Full thickness	21 (42%)
Labral tear	Any (grade 2–5 in any region)	44 (88%)
BML	Any (grade 1–3 in any region)	5 (10%)
Subchondral cyst	Any (grade 1–2 in any region)	5 (10%)
Ligamentum teres tear	Any (grade 2 or above)	0 (0%)
Paralabral cyst		12 (24%)
Loose bodies		0 (0%)
Effusion-synovitis		0 (0%)

*BML* bone marrow lesion

**Table 7:** Baseline and delta (change in sum score between baseline and 2-year follow-up) OA feature sum (total) scores *n* = 50 hips

SHOMRI feature	Median (IQR)
Baseline cartilage sum score	2 (1, 4)
Baseline labral sum score	5 (3, 7)
Baseline BML sum score	0 (0, 0)
Baseline subchondral cyst sum score	0 (0, 0)
Delta cartilage sum score	0 (0, 2)
Delta labral sum score	2 (1, 3)
Delta BML sum score	0 (0, 0)
Delta subchondral cyst sum score	0 (0, 0)

*BML* bone marrow lesion

### SHOMRI feature change over time

Change in SHOMRI feature sum scores (baseline to 2 years) is presented in Table 7. Cartilage defect worsening was evident in 48% of hips (Supplementary information). The number of new subregions affected by partial- and full-thickness defects ranged from − 2 to 3 and 0–2, respectively. Close to half of all hips (44%) had a new labral tear in one or more subregion, and 78% of hips had labral tear worsening in one or more subregion (Supplementary information). The maximum change in labral score ranged from 1 to 4. For BMLs, the change in number of subregions affected ranged from − 2 (two fewer subregions affected by BMLs) to 1 (one more subregion affected by BMLs) (Supplementary information). Five hips (10%) had evidence of BML worsening, with only one hip showing improvement. Four hips (8%) had one new subregion affected by a subchondral cyst, with improvement not observed in any hips (Supplementary information). Of the remaining features, only ligamentum teres tears and paralabral cysts affected more hips at follow-up than baseline (Supplementary information).

## Discussion

This study extends the original technical study of the SHOMRI grading system by providing definitions for reporting feature prevalence, severity, and change. This is critical to enable use of this grading system in a clinical context to analyze structural OA disease development and progression. A high prevalence of cartilage defects and labral tears was observed in football players with hip and/or groin pain, with up to 78% of hips demonstrating MRI feature worsening over 2 years, highlighting the sensitivity of SHOMRI to assess incidence and progression of degenerative changes.

Variability in the reporting of OA feature prevalence and severity has been highlighted in two recent systematic reviews [17, 18]. To overcome this, we provide comprehensive definitions for eight OA features that will enable consistent reporting in future studies using the SHOMRI scoring tool. Comprehensive evaluation of OA feature severity is challenging, but important for understanding OA pathogenesis and the link between structural change and symptoms. Existing studies often do not report OA feature severity across the whole joint (i.e., number of subregions), opting for total or sum scored instead [8, 10]. Our definitions for severity overcome this deficiency by providing both measures, affording a comprehensive assessment of severity.

Our proposed change definitions permit SHOMRI feature improvement and worsening, but not all features were allowed to improve. For example, cartilage defects, labral tears, and ligamentum teres tears were not allowed to improve over time. This approach is consistent with semi-quantitative change definitions for knee OA [12]. MRI improvement of tissue morphology (without surgical repair) may represent the formation of scar tissue rather than normal tissue regeneration (i.e., healing) [19, 20]. We acknowledge that our definitions may not be suitable for clinical trials evaluating the efficacy of disease-modifying treatments. Bone marrow lesions and subchondral cysts are dynamic features of OA disease, with the ability to fluctuate in size over time [12, 21]. Improvement and worsening of BMLs and subchondral cysts were allowed in our change definition, as in definitions for longitudinal change in studies of knee OA [11, 12].

Longitudinal change of hip MRI features has yet to be studied in detail [22]. In the present study, we propose several measures to thoroughly describe the spectrum of structural OA change. Summed scores have drawn criticism as they represent heterogeneous change in features [11, 12]. For instance, a summed cartilage change score of 6 could be achieved through three incident full-thickness defects or six incident partial-thickness defects, which are arguably different disease states. However, with knowledge of their shortcomings and when used alongside approaches that capture change within the entire joint, summed scores still provide a sensitive measure for monitoring change in OA features. Change across the entire joint was captured through evaluation of subregions. This approach overcomes the deficiencies of summed scores and permits assessment of change in existing features and the identification of incident pathology [12]. We also include a max change score across the entire hip, providing a measure of overall change that can be missed with summed scores [12]. Further work is needed to understand if specific change measures are related to symptom worsening and hip OA development.

Using our proposed definitions, a large proportion of symptomatic adult football players had hip OA features and demonstrated worsening of these features over 2 years. The reported prevalence of the eight OA features is largely consistent with existing investigations of similar populations [17, 18]. However, the extent of worsening in key hip OA features including labral tears (78% vs 17%) and cartilage defects (48% vs 11%) was much higher than previously reported in older non-athletic subjects [10]. As both studies used similar SHOMRI feature change definitions, differences likely reflect variations in participant characteristics (e.g., hip morphology, physical activity) and the complex nature of hip OA development [23–25].

Examination of MRIs was completed with knowledge of MRI sequences and timepoint but not clinical status. This approach is recommended for longitudinal investigations as it improves sensitivity to feature change [3, 11]. A consensus process was used to determine the final grading for each MRI feature. While this may take additional time to complete, it simplifies the reporting of features by providing a single grading for both readers.

We recognize there are several limitations that require consideration when using the proposed definitions. There is considerable debate surrounding the ability of MRI-defined OA features to improve over time [11, 12]. As our understanding of hip OA pathogenesis evolves, we recognize that revision of the proposed definitions may be needed to optimize the reporting of feature change. We used an optimized 3 T MRI protocol for assessment hip OA features. We acknowledge that contrast-enhanced MRI may provide superior assessment of key OA features, including cartilage, labrum, and synovium [26–29]. However, the use of contrast-enhanced MRI in longitudinal studies is associated with risk and not appropriate in all symptomatic populations. The binary classification of effusion-synovitis is likely to be insensitive to longitudinal change. This deficiency may preclude us from understanding the importance of effusion-synovitis in OA progression.

This study is the first to provide rigorous criteria and definitions for reporting prevalence, severity, and change of hip degenerative change using the SHOMRI grading system. Using the proposed definitions, up to 78% of hips demonstrated feature change over 2 years, well demonstrating sensitivity to change. Use of our definitions will enable comparison between hip MRI studies in clinical cohorts and improve our understanding of hip OA pathogenesis and progression. Longitudinal studies are now required to provide insight into the prognostic implications of the proposed SHOMRI definitions.

### Supplementary Information

Below is the link to the electronic supplementary material.

Supplementary file1 (PDF 291 KB)
