# Supplementary material for: Defining hip osteoarthritis feature prevalence, severity, and change using the Scoring of Hip Osteoarthritis with MRI (SHOMRI)
Source: Skeletal Radiol. 2024 Mar 9;53(8):1599–609. doi: 10.1007/s00256-024-04628-0 (PMC11194192; doi:10.1007/s00256-024-04628-0)
Supplement: Supplementary file 1 — Supplementary file1 (PDF 291 KB) [file 256_2024_4628_MOESM1_ESM.pdf]

Supplementary information

**Supplementary Table 1**. Baseline – Cartilage defect characteristics (n=50 hips)		
SHOMRI feature	Outcome	Number of hips, n (%)
Cartilage defect		
	Cartilage defect location (partial-thickness)	
	Anterior (acetabulum)	3 (6%)
	Anterior (femur)	0 (0%)
	Posterior (acetabulum)	2 (4%)
	Posterior (femur)	1 (2%)
	Superolateral (acetabulum)	15 (30%)
	Superolateral (femur)	15 (30%)
	Superomedial (acetabulum)	11 (22%)
	Superomedial (femur)	5 (10%)
	Lateral (femur)	8 (16%)
	Inferior (femur)	2 (4%)
	Acetabulum any region	23 (46%)
	Femur any region	19 (38%)
	Cartilage defect location (full-thickness)	
	Anterior (acetabulum)	0 (0%)
	Anterior (femur)	0 (0%)
	Posterior (acetabulum)	2 (4%)
	Posterior (femur)	3 (6%)
	Superolateral (acetabulum)	3 (6%)
	Superolateral (femur)	2 (4%)
	Superomedial (acetabulum)	7 (14%)
	Superomedial (femur)	0 (0%)
	Lateral (femur)	11 (22%)
	Inferior (femur)	2 (4%)
	Acetabulum any region	10 (20%)
	Femur any region	16 (32%)

Number of subregions affected by any cartilage defect	
0	12 (24%)
1	16 (32%)
2	2 (4%)
3	10 (20%)
4	8 (16%)
5	2 (4%)
6	0 (0%)
7	0 (0%)
8	0 (0%)
9	0 (0%)
10	0 (0%)
Number of subregions affected by partial-thickness cartilage defects	
0	19 (38%)
1	15 (30%)
2	4 (8%)
3	9 (18%)
4	3 (6%)
5	0 (0%)
6	0 (0%)
7	0 (0%)
8	0 (0%)
9	0 (0%)
10	0 (0%)
Number of subregions affected by full-thickness cartilage defects	
0	29 (58%)
1	14 (28%)
2	5 (10%)
3	2 (4%)

4	0 (0%)
5	0 (0%)
6	0 (0%)
7	0 (0%)
8	0 (0%)
9	0 (0%)
10	0 (0%)
Max cartilage score hip (all regions)	
0	12 (24%)
1	17 (34%)
2	21 (42%)
Summed cartilage score	
0	12 (24%)
1	11 (22%)
2	5 (10%)
3	5 (10%)
4	5 (10%)
5	8 (16%)
6	3 (6%)
7	0 (0%)
8	1 (2%)

**Supplementary Table 2**. Baseline - labral tear characteristics (n=50 hips)		
SHOMRI feature	Outcome	Number of hips, n (%)
Labral tear		
	Severity	
	No labral tear	6 (12%)
	Simple (grade 2 or 3 in any region)	27 (54%)
	Severe (grade 4 or 5 in any region)	17 (34%)
	Labral tear location (Any)	
	Anterior	9 (18%)
	Anterosuperior	24 (48%)
	Superior	34 (68%)
	Posterior	11 (22%)
	Number of subregions affected by any labral tear (grade 2-5)	
	0	6 (12%)
	1	18 (36%)
	2	18 (36%)
	3	8 (16%)
	4	0 (0%)
	Max labral score hip (all regions)	
	0	2 (4%)
	1	4 (8%)
	2	5 (10%)
	3	22 (44%)
	4	12 (24%)
	5	5 (10%)
	Summed labral score	
	0	2 (4%)
	1	0 (0%)
	2	5 (10%)
	3	8 (16%)
	4	6 (12%)

5	9 (18%)
6	7 (14%)
7	1 (2%)
8	1 (2%)
9	3 (6%)
10	1 (2%)
11	6 (12%)
12	0 (0%)
13	1 (2%)

Supplementary Table 3. Baseline – BML characteristics (n=50 hips)

SHOMRI feature	Outcome	Number of hips, n (%)
BMLs		
	BML location	
	Anterior (acetabulum)	1 (2%)
	Anterior (femur)	0 (0%)
	Posterior (acetabulum)	1 (2%)
	Posterior (femur)	0 (0%)
	Superolateral (acetabulum)	3 (6%)
	Superolateral (femur)	0 (0%)
	Superomedial (acetabulum)	1 (2%)
	Superomedial (femur)	0 (0%)
	Lateral (femur)	0 (0%)
	Inferior (femur)	0 (0%)
	Acetabulum any region	6 (12%)
	Femur any region	0 (0%)
	Number of subregions affected by any BML	
	0	45 (90%)
	1	4 (8%)
	2	2 (2%)
	Max BML score hip (all regions)	
	0	45 (90%)
	1	3 (6%)
	2	2 (4%)
	Summed BML score	
	0	45 (90%)
	1	2 (4%)
	2	3 (6%)

Abbreviations: BML, bone marrow lesion

Supplementary Table 4. Baseline – subchondral cyst characteristics (n=50 hips)

SHOMRI feature	Outcome	Number of hips, n (%)
Subchondral cyst		
	Subchondral cyst location	
	Anterior (acetabulum)	1 (2%)
	Anterior (femur)	0 (0%)
	Posterior (acetabulum)	0 (0%)
	Posterior (femur)	0 (0%)
	Superolateral (acetabulum)	3 (6%)
	Superolateral (femur)	0 (0%)
	Superomedial (acetabulum)	1 (2%)
	Superomedial (femur)	0 (0%)
	Lateral (femur)	0 (0%)
	Inferior (femur)	0 (0%)
	Acetabulum any region	5 (10%)
	Femur any region	0 (0%)
	Number of subregions affected by any subchondral cyst	
	0	45 (90%)
	1	5 (10%)
	Max subchondral cyst score hip (all regions)	
	0	45 (90%)
	1	4 (8%)
	2	1 (2%)
	Summed subchondral cyst score	
	0	45 (90%)
	1	4 (8%)
	2	1 (2%)

**Supplementary Table 5**. Change in cartilage defects baseline to 2-years (n=50 hips)		
SHOMRI feature	Outcome	Number of hips, n (%)
Cartilage defect		
	Number of new subregions with cartilage defect (any)	
	0	31 (62%)
	1	13 (26%)
	2	3 (6%)
	3	3 (6%)
	Number of new subregions with partial-thickness cartilage defect	
	-2*	1 (2%)
	-1*	4 (8%)
	0	32 (64%)
	1	9 (18%)
	2	1 (2%)
	3	3 (6%)
	Number of new subregions with full-thickness cartilage defect	
	0	37 (74%)
	1	12 (24%)
	2	1 (2%)
	Number of subregions with worsening of cartilage defect	
	0	26 (52%)
	1	15 (30%)
	2	5 (10%)
	3	4 (8%)
	Worsening in any subregion	
	No	26 (52%)
	Yes	24 (48%)
	Max change in cartilage score in all regions	

0	41 (82%)
1	8 (16%)
2	1 (2%)
Change in summed cartilage score (all regions combined)	
0	26 (52%)
1	11 (22%)
2	8 (16%)
3	5 (10%)
Worsening (total score increase>0)	
No	26 (52%)
Yes	24 (48%)

*hips progressed from partial thickness to full thickness defects over 2-years

**Supplementary Table 6**. Change in labral tears baseline to 2-years (n=50 hips)		
SHOMRI feature	Outcome	Number of hips, n (%)
Labral tear		
	Number of new subregions with labral tear (any)	
	0	28 (56%)
	1	18 (36%)
	2	3 (6%)
	3	1 (2%)
	4	0 (0%)
	Number of subregions with worsening of labral tear	
	0	11 (22%)
	1	20 (40%)
	2	13 (26%)
	3	5 (10%)
	4	1 (2%)
	Worsening in any subregion	
	No	11 (22%)
	Yes	39 (78%)
	Max change in labral score in all regions	
	0	11 (22%)
	1	14 (28%)
	2	17 (34%)
	3	5 (10%)
	4	3 (6%)
	5	0 (0%)
	Change in summed labral score (all regions combined)	
	0	11 (22%)
	1	9 (18%)
	2	11 (22%)

3	12 (24%)
4	3 (6%)
5	1 (2%)
7	2 (4%)
9	1 (2%)
Worsening (total score increase>0)	
No	11 (22%)
Yes	39 (78%)

Supplementary Table 7. Change in BMLs baseline to 2-years (n=50 hips)

SHOMRI feature	Outcome	Number of hips, n (%)
BML		
	Change in number of subregions with BML	
	-2	1 (2%)
	0	47 (94%)
	1	2 (4%)
	Number of subregions with worsening of BML	
	0	45 (90%)
	1	5 (10%)
	Worsening in any subregion	
	No	45 (90%)
	Yes	5 (10%)
	Number of subregions with improvement of BML	
	0	49 (98%)
	1	0 (0%)
	2	1 (2%)
	Improvement in any subregion	
	No	49 (98%)
	Yes	1 (2%)
	Max change in BML score in all regions	
	-1	1 (2%)
	0	44 (88%)
	1	3 (6%)
	2	1 (2%)
	3	1 (2%)
	Change in summed BML score (all regions combined)	
	-2	1 (2%)
	0	44 (88%)

1	3 (6%)
2	1 (2%)
3	1 (2%)
Worsening (increase in total score >0)	
No	45 (90%)
Yes	5 (10%)

Supplementary Table 8. Change in subchondral cysts baseline to 2-years (n=50 hips)

SHOMRI feature	Outcome	Number of hips, n (%)
Subchondral cysts		
	Change in number of subregions with subchondral cysts	
	0	46 (92%)
	1	4 (8%)
	Number of subregions with worsening of subchondral cysts	
	0	46 (92%)
	1	4 (8%)
	Worsening in any subregion	
	No	46 (92%)
	Yes	4 (8%)
	Improvement in any subregion	
	No	50 (100%)
	Yes	0 (0%)
	Max change in subchondral cyst score in all regions	
	0	46 (92%)
	1	3 (6%)
	2	1 (2%)
	Change in summed subchondral cysts score (all regions combined)	
	0	46 (92%)
	1	3 (6%)
	2	1 (2%)
	Worsening (increase in total score >0)	
	No	46 (92%)
	Yes	4 (8%)

Supplementary Table 9. 2-year – hip OA feature prevalence (n=50 hips)

SHOMRI feature	Outcome	Number of hips, n (%)
Ligamentum teres tears	Any (grade 2 or above)	
		1 (2%)
Paralabral cysts		
		16 (32%)
Loose bodies		
		0 (0%)
Effusion-synovitis		
		0 (0%)
